# Genome editing

**DOI:** 10.1038/s41598-022-24850-x

**Published:** 2022-11-28

**Authors:** Maura McGrail, Tetsushi Sakuma, Leonidas Bleris

**Affiliations:** 1 Department of Genetics, Development and Cell Biology, Iowa State University, Ames, IA USA; 2 Division of Integrated Sciences for Life, Graduate School of Integrated Sciences for Life, Hiroshima University, Hiroshima, Japan; 3 Bioengineering Department, The University of Texas at Dallas, Dallas, TX USA

**Keywords:** Mutagenesis, Epigenomics

## Abstract

Recent advances in genome editing technologies have redefined our ability to probe and precisely edit the human genome and epigenome in vitro and in vivo*.* More specifically, RNA-guided CRISPR/Cas systems have revolutionized the field due to their simplicity in design and adaptability across biological systems. This Collection highlights results in CRISPR/Cas technology that increase the efficiency of precision genome editing, and allow genetic manipulation in model systems traditionally intractable to site-directed gene modification.

The development of custom Cas9 endonucleases for CRISPR/Cas9 genome editing has revolutionized the field of genetics and functional genomics^1–3^. CRISPR/Cas9 targeting of a DNA double strand break provides a template for cellular repair mechanisms that introduce mutations through error-prone Non-Homologous End Joining, or allow precision knock-in of exogenous sequences by Homology Directed Repair^4–11^. The utility of CRISPR/Cas9 editing has been greatly expanded by engineering of Cas9 variants with single strand nicking activity to reduce off-target genome edits^6^, altered protospacer adjacent motif (PAM) recognition sequences to broaden the targetable genome^12–15^, and inactivated enzymes which lack endonuclease activity (dCas9)^16^. dCas9 variants create a platform for generation of fusion proteins with novel gene editing function, including epigenome editing through regulation of histone modifications^17–21^, deaminases that modify nucleotide sequences through base editing^22,23^, and reverse transcriptase which introduces genome edits from an RNA template through prime editing^24^. These powerful Cas9-based genome editing tools allow targeted modification of nearly any gene of interest across most biological systems, enabling molecular genetic studies in organisms in which gene manipulation and transgenesis has traditionally been difficult^25,26^. Key considerations in the application of this technology are optimal delivery and expression of CRISPR/Cas9 reagents.

This Collection gathers 17 contributions describing new methods to improve CRISPR/Cas9 genome editing efficiency in a variety of animal and plant systems^27–34^, and its application to develop new genetic tools and models^35–38^ and to gain novel insight into mechanisms regulating organismal physiology, cell behavior, and gene expression^39–43^.

The relatively low efficiency of CRISPR/Cas9 targeted integration of exogenous DNA via HDR has been a limiting factor in precision knock-in in mammalian cells and in vivo systems in which the predominant DNA repair pathway is Non-Homologous End Joining (NHEJ). Optimization of each parameter in CRISPR knock-in experimental design, including the specific Cas endonuclease, single or double-stranded DNA template, linear or circular templates, and homology arm length increases the chances of recovering precision HDR edits. For gene editing in mammalian cells using single-stranded oligonucleotide templates, guide RNA selection, donor strand preference, and introduction of blocking mutations in the template to prevent cleavage, together lead to significantly higher frequencies of on target precision integration^27^. Experimental manipulation to enhance HDR knock-ins involve altering the relative activity of different DNA repair pathways by inhibition of the NHEJ DNA repair enzymes DNA Polymerase θ and DNA-PK, in combination with in vivo template liberation^28^.

An important consideration in optimizing gene editing experimental design is the need for efficient delivery and robust expression of CRISPR/Cas9 editing reagents in individual systems. Replacement of *Drosophila* gene promoters in CRISPR/Cas9 targeting plasmids with *Aedes aegypti* counterparts lead to high level Cas9 and gRNA gene expression, and the efficient recovery of knock-in edited mosquito cell lines expressing Flag-tagged *AGO1* for future functional studies^30^. Use of an endogenous RPS5a promoter for expression of deaminase base editors in *Arabidopsis thaliana* increased base editing efficiency > 30% in comparison to the heterologous CaMV35S viral promoter^34^. These studies underscore species-specific gene regulatory elements may be required for optimal gene editing in some systems. Generating genetically modified mice by gene editing was shown to be facilitated by an Integrated Automated Embryo Manipulation System, that positions mouse embryos for reproducible pronuclear microinjection^31^. Optimal injection conditions allowed efficient introduction of gene edits and increased embryo survival, enhancing the recovery of genome edited adult mice. In difficult to transfect cells such as human iPSCs, a piggyBac transposon system provided sustained expression of Cas9 prime editors, a critical factor when combined with optimal pegRNA template design, for efficient gene editing^29^. Recovery of patient derived CRISPR-Cas9 knockout leukemia cells was significantly improved by the development of a fluorescent reporter system that provides a readout of cells harboring high levels of gene editing^38^.

The Cas9 targetable genome has been significantly expanded with the development of near-PAM-less Cas9 variants that recognize NG, NA, NT and NC PAMs. In *Dictyostelium discoidium* cells near-PAM-less Cas9 variants were compared and shown to promote efficient knock-in of a double-stranded fluorescence reporter template or single-stranded oligos harboring single nucleotide changes^32^. Application of genome editing in *D. discoidium*, an in vivo system for investigating molecular mechanisms in the evolution of multi-cellular organisms, now allows investigators to examine the genetic requirements for multicellular communication and coordinated cell–cell interactions. As an alternative to Cas9-induced double strand breaks to stimulate HDR, tandem paired nicking using Cas9 D10A nickases, with long homology templates of 1700–2000 bp and an optimal 20 nucleotide gRNA length, drove efficient precise knock-in in HCT116 cells^33^, suggesting the potential for applying TPN targeting in other cell types.

CRISPR-directed knock-in and knock-out have been widely applied to generate novel genetic tools and models to investigate gene function. The CRISPR/Cas9 GeneWeld short homology arm knock-in approach for generating targeted integrations^44^ was used to integrate Cre recombinase into zebrafish proneural genes, placing Cre expression under the control of endogenous gene regulatory elements to increase the specificity and reproducibility of Cre recombinase activity for improved cell lineage labeling and conditional gene studies^35^. Application of PITCh (Precise Integration into Target Chromosome)^45^ to target the Ca2 + sensor GCaMP3 into the midge *Polypedilum vanderplanki* Pv11 cells suggests an important role for calcium signaling in anhydrobiosis in midge larvae^40^. HDR has also been used to increase the robustness of PD-L1 gene knock-down in cultured glioblastoma cells, by introducing a template harboring a stop codon, revealing a critical role for PD-L1 in preventing proliferation, invasion and tumor associated macrophage polarization to M1 phenotype^39^.

The high efficiency of CRISPR knockout with single or dual gRNAs targeting exons has generated multiple new genetic models including investigation of the role of exon skipping in frame-shift mutant alleles in mice^36^, the role of HOL methyltransferases in methyl iodide emission from rice *Oryza sativa*^41^, and patient derived cultured myoblast models of Duchenne muscular dystrophy for chemical screens^37^. CRISPR/Cas9-guided promoter deletion is also an effective method for gene knock-down as shown by H19 lncRNA knock-out impacting cell proliferation and genome stability^42^. Double strand and single strand break-free methods for silencing gene expression using epigenome editors allows direct demonstration of the relationship between H3K27Ac in gene promoter leading to H3K4me3 enrichment^43^, providing an elegant, non-mutagenic approach to investigate crosstalk in epigenetic mechanisms regulating transcriptional activation.

The papers published in this Collection highlight how optimal experimental design and development of species specific reagents enhance the efficiency of CRISPR/Cas9 genome editing. These advances illustrate the tremendous power of CRISPR/Cas9 genome editing to open new areas of investigation and allow genetic manipulation in model systems traditionally intractable to site-directed gene modification.
